# Febrile "migrating" eosinophilic cellulitis with hepatosplenomegaly: adult toxocariasis – a case report

**DOI:** 10.1186/1757-1626-1-356

**Published:** 2008-11-28

**Authors:** Ioannis D Bassukas, Georgios Gaitanis, Aikaterini Zioga, Christina Boboyianni, Christina Stergiopoulou

**Affiliations:** 1 Dept. of Skin & Venereal Diseases, Univ. Ioannina Medical School and Univ. Hospital, Ioannina, Greece; 2 Dept. of Pathology, Univ. Ioannina Medical School and Univ. Hospital, Ioannina, Greece; 3 Dept. of Clinical Microbiology; Univ. Ioannina Medical School and Univ. Hospital, Ioannina, Greece

## Abstract

**Background:**

Eosinophilic cellulitis (Wells' syndrome) is a polyetiologic clinical entity with still obscure pathogenesis. Clinically overt toxocariasis is uncommon in adults, yet helminthozoonoses, including toxocariasis have been occasionally implicated in the pathogenesis of eosinophilic cellulitis.

**Case representation:**

A 55-year-old female patient presented with a skin biopsy verified recurring febrile eosinophilic cellulitis, blood eosinophilia (42%), slight anaemia (Hct 35%), hepatosplenomegaly and positive specific anti-*Toxocara canis *antibodies. Toxocariasis-associated eosinophilic cellulitis was diagnosed. Already two weeks after treatment with thiabendazole the skin lesions resolved, *T. canis *antibody titre normalized eight months after treatment and no recurrences of eosinophilic cellulitis have been observed (for meanwhile three years).

**Conclusion:**

The clinical characteristics (relapsing skin lesions, fever, hepatosplenomegaly), the laboratory features (blood eosinophilia, modest anemia, positive *T. canis *serology) and the clinical course after treatment, all support a causal relationship between *Toxocara *infection and the disease of this patient. We propose that in this context eosinophilic cellulitis must be interpreted as the leading symptom of a "skin-predominant" form of overt adult toxocariasis out of a spectrum of toxocariasis-associated febrile, "migrating-relapsing", organotropic eosinophilic inflammatory syndromes.

## Background

Eosinophilic cellulitis (Wells' syndrome) is an established, polyetiologic clinical entity with still obscure pathogenesis. The oedematous erysipelas- or urticaria-like plaques of eosinophilic cellulitis appear acutely and later develop into morphea-like, slate blue colored indurations, fading slowly over weeks to months [1,2]. Distinct, though not pathognomonic is the characteristic eosinophilic inflammation of the skin presenting with "flame figures" at histopathologic sections [2]. Blood (and bone marrow) eosinophilia, although not consistently found, is a significant diagnostic criterion too [2].

Helminthozoonoses, including toxocariasis, have been implicated in the pathogenesis of eosinophilic cellulitis [3-6]. Toxocariasis is a cosmopolitic endoparasitosis, caused by *Toxocara *species (*T. canis *and *T. cati*), the roundworms of various carnivores [7]. In the aberrant human host larvae hatch from ingested viable eggs in the proximal intestine, enter the circulation and wander through the body. Clinically evident toxocariasis in adults is rare. Its diagnosis is often based on a constellation of suggestive clinical signs and laboratory findings, like eosinophilia, positive serology and outcome after antihelminthic treatment rather than on pathognomonic clinical pictures [7].

Here on the occasion of a case of a toxocariasis in an adult patient, which presented as eosinophilic cellulitis we discuss the evidence that some cases of febrile, "migrating-relapsing" organotropic eosinophilic inflammations, like eosinophilic cellulitis, may consist a nosologic family of clinical manifestation syndromes of toxocariasis in the adult.

## Case presentation

A 55-year-old female patient was referred to the Dermatology Department with erythematous, infiltrating plaques of the lower extremities and concurrent relapsing fever up to 38,5°C of two-months duration. The patient had already received several courses of antibiotic treatments (penicillin/cefuroxim plus ciprofloxacin) for "recurrent erysipelas" without substantial improvement. She was otherwise healthy, with unremarkable medical history and without receiving any medication.

**Figure 1:**
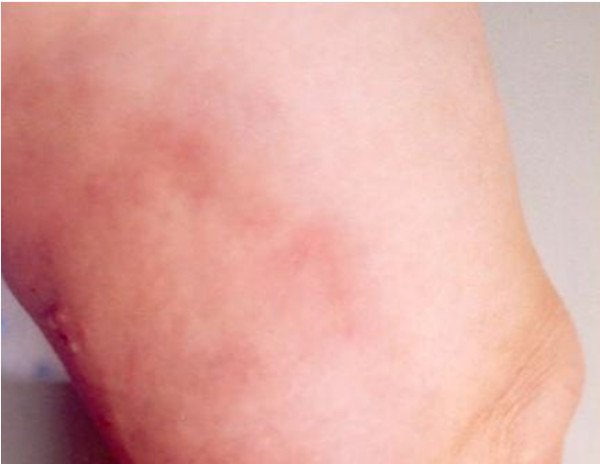
**Clinical presentation of acute phase eosinophilic cellulitis**. Inflammatory, edematous plaque at the left popliteal region.

**Figure 2:**
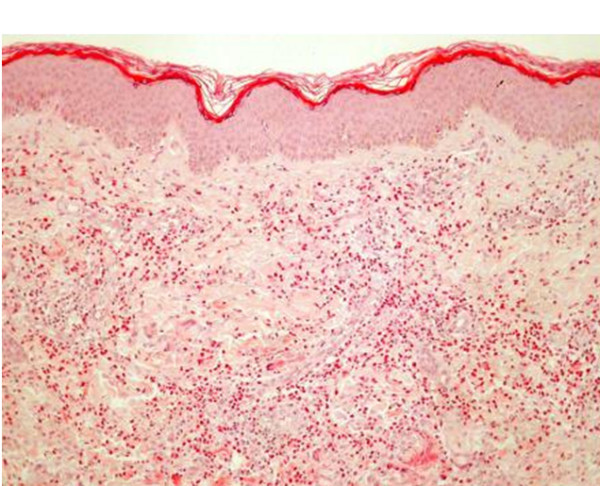
**Histology of skin biopsy from acute phase eosinophilic cellulitis. Note findings characteristic of early phase eosinophilic cellulitis**. Plentiful tissue eosinophils and flame figures at the deeper chorium sections (hematoxylin & eosin, original magnification ×40).

On admission she showed a two-month-old morphea-like lesion at her right thigh and a recent inflammatory plaque at left popliteal region/left distal thigh (Figure 1). Morphea and EC were considered as differential diagnoses. Skin biopsies of both lesions revealed histologic findings characteristic of late and early phase EC correspondingly (Figure 2). Laboratory evaluation showed blood eosinophilia (44.2% of total WBC, absolute number of eosinophils 2930 cells/μl) and modest anaemia (Hct 35%, Hb 11,5 g/dl). The CRP, ASOT, ESR, serum tumor markers (alphafetoprotein α-FP, carcinoembryonic antigen CEA, Ca 19-9, Ca 15-3, Ca 125), routine liver and renal function tests, routine urine analysis were all within the physiological range. Increased titer of specific IgG antibodies (patient's sample to control ratio = 1.82) against *Toxocara canis *were determined with a commercial ELISA kit (Cypress Diagnostics, Langdorp, Belgium; test diagnostic for *Toxocara *infection for titer-ratio ≥ 1.10). Stool microscopy was negative for parasites or parasitic eggs. Chest X ray, brain CT (to exclude subclinical CNS involvement) and duplex ultrasound of the lower extremity veins were unremarkable. Abdominal ultrasound revealed modest hepatosplenomegaly. The patient lived in a rural area and reported housing several dogs.

The diagnosis of toxocariasis (visceral larva migrans) presenting as EC was suspected and two courses, four days apart, of oral thiabendazole (Mintezol, Merck: 3 × 500 mg b.i.d for two days) were conducted. Two weeks latter skin lesions resolved and on the occasion of a follow-up examination eight months later, *T. canis *antibody titer normalized too. The patient remains relapse-free for meanwhile 3 years without serological evidence of reinfection.

## Discussion

The key event in the pathogenesis of eosinophilic cellulitis appears to be a sustained activation of eosinophils as a result of some aberrant regulation of the inflammatory process in the skin leading to tissue destruction with formation of the well-known, diagnostic, though not characteristic, "flamme figures" in routine histology [8]. According to the leading pathogenetic hypothesis in the literature eosinophilic cellulitis represents a "non-specific" hypersensitivity tissue reaction pattern of the skin to different triggering factors such as infections, drugs, or internal diseases including malignancies [9]. The alternative hypothesis predicts that eosinophilic cellulitis may be the result of sustained, uncontrolled activation of eosinophils at the site of an initially urticarial skin lesion, probably as the result of the potentiation of a Th_2_-inflammatory response, which occurs upon exposure to a particular antigen during the initiation of another Th_2 _response from another stimulus, like infection with a parasite [10]. Likewise besides urticaria other Th_2_-depended primary skin lesions may stay at the start of eosinophilic cellulitis too. Eosinophils enter target tissues in a resting state and in order to become activated must be first primed by exposure to key-cytokines. Increased levels of serum interleukin-5 (IL-5), the essential cytokine for eosinophil activation, fluctuating according to disease severity, have been described in a case of eosinophilic cellulitis and activated CD4^+^CD7^- ^Th_2 _lymphocytes were considered responsible for its release [11]. Eosinophils might further cause tissue fibrosis and produce the morphea-like lesions of late eosinophilic cellulitis through the production of cytokines with relevant action, like members of the TGF, EGF, and PDGF families [12].

Eosinophils are well-known to be the effector cells for direct killing metazoans and the invasion of an immunocompetent host by metazoan parasites, like *Toxocara canis*, may induce severe blood and tissue eosinophilia [13]. Ingested *Toxocara *ova hatch in the intestine and the second-stage larvae migrate to other organs (liver, lungs, muscles, brain, eyes), where they may induce eosinophilic granulomas, an extensive cellular infiltrate of eosinophils, neutrophils and lymphocytes [14]. However, in spite of clear evidence for eosinophilia, mastocytosis and elevated IgE levels in many helminthic human diseases little sign of hypersensitivity do exist, possibly as the result of balancing homeostatic mechanisms between immunity and limitation of tissue damage from uncontrolled activation of inflammatory processes [15]. In accordance to this general rule most *Toxocara *infestations in adults remain usually asymptomatic (covert toxocariasis), although young children may develop symptomatic visceral larva migrans and more rarely ocular granulomas (ocular larva migrans) [7]. Migrating *Toxocara *larvae have not yet been demonstrated in skin sections. However, the skin has been implicated in cases of toxocariasis with the development of generalized pruritus, urticaria and prurigo [7,16], eosinophilic cellulitis [6], eosinophilic folliculitis [17] and vasculitis [18]. Quite exceptional, though perhaps pathognomonic for toxocariasis, is a migrating eosinophilic panniculitis characterized by the eruption of tender, subcutaneous nodules, lasting 1–2 weeks [19].

## Conclusion

Wells and Smith [1], on the basis of theoretical pathophysiological considerations, were the first to suggest a possible association of EC with toxocariasis and indeed, some years later Hunri et al [6] presented direct clinical evidence favouring this connection. The present case further corroborates the addition of some EC cases into the spectrum of adult toxocariasis clinical syndromes. However, the clinical characteristics of the present patient (relapsing skin lesions, fever and hepatosplenomegaly) together with the laboratory features (blood eosinophilia, modest anemia and positive *Toxocara *serology) support the suggestion that the eosinophilic cellulitis in cases like the present one must be interpreted as the leading symptom of a distinct syndrome of symptomatic adult toxocariasis. Evidence in the literature suggests that different organs are differently affected by activated eosinophils and they may further respond in a distinct fashion to the action of IL-5 [12]. Future studies should evaluate whether a subset of "migrating eosinophilic inflammations" in adults presenting as organotropic inflammations to different organs may be the common manifestation form of a toxocariasis-associated spectrum of "eosinophilic" syndromes, including, at the present time, eosinophilic arthritis [7], eosinophilic pleuritis [20], eosinophilic panniculitis [19] and eosinophilic cellulitis ([6] and present case).

## Consent

Written informed consent was obtained from the patient for publication of this case report and accompanying image. A copy of the written consent is available for review by the Editor-in-Chief of this journal.

## Competing interests

The authors declare that they have no competing interests.

## Authors' contributions

IDB analyzed and interpreted the patient data and was the major contributor in writing the manuscript. GG was a major contributor in writing the manuscript. AZ performed the histological examination and interpretation. CB performed the serological examination and interpretation. CS acquired the patient data and contributed to the writing of the manuscript. All authors read and approved the final manuscript.

## References

[B1] Wells GC, Smith NP (1979). Eosinophilic cellulitis. Br J Dermatol.

[B2] Aberer W, Konrad K, Wolff K (1988). Wells' syndrome is a distinctive disease entity and not a histologic diagnosis. J Am Acad Dermatol.

[B3] Hoogenband HM Van der (1983). Eosinophilic cellulitis as a result of onchocerciasis. Clin Exp Dermatol.

[B4] Prendiville JS, Jones RR, Bryceson A (1985). Eosinophilic cellulitis as a manifestation of onchocerciasis. J R Soc Med.

[B5] Tsuda S, Tanaka K, Miyasato M, Nakama T, Sasai Y (1994). Eosinophilic cellulitis (Wells' syndrome) associated with ascariasis. Acta Derm Venereol.

[B6] Hunri MA, Gerbig AW, Braathen LR, Hunzinker T (1997). Toxocariasis and Wells' syndrome: a causal relationship?. Dermatology.

[B7] Despommier D (2003). Toxocariasis: clinical aspects, epidemiology, medical ecology, and molecular aspects. Clin Microbiol Rev.

[B8] Brehmer-Andersson B, Kaaman T, Skog B, Frithz A (1986). The histopathogenesis of the flame figure in Wells' syndrome based on five cases. Acta Derm Venereol.

[B9] Mitchell AJ, Anderson TF, Headington JT, Rasmussen JE (1984). Recurrent granulomatous dermatitis with eosinophilia. Wells' syndrome. Int J Dermatol.

[B10] Wells GC (1971). Recurrent granulomatous dermatitis with eosinophilia. Trans St Johns Hosp Dermatol Soc.

[B11] España A, Sanz ML, Sola J, Gil P (1999). Wells' syndrome (eosinophilic cellullitis): correlation between clinical activity, eosinophil levels, eosinophil cation protein and interleukin-5. Br J Dermatol.

[B12] Wardlaw AJ (1999). Molecular basis for selective eosinophil trafficking in asthma: a multistep paradigm. J Allergy Clin Immunol.

[B13] Parsons JC, Bowman DD, Grieve RB (1989). Pathological and haematological responses of cats experimentally infected with *Toxocara canis *larvae. Int J Parasitol.

[B14] Kusama Y, Takamoto M, Kasahara T, Takatsu K, Nariuchi H, Sugane K (1995). Mechanisms of eosinophilia in BALB/c-*nu */+ and congenitally athymic BALB/c-*nu*/*nu *mice infected with *Toxocara canis*. Immunology.

[B15] Allen JE, Maizels RM (1996). Immunology of human helminth infection. Int Arch Allergy Immunol.

[B16] Humbert P, Niezborala M, Salembier R, Aubin F, Piarroux R, Buchet S, Barale T (2000). Skin manifestations associated with toxocariasis: a case-control study. Dermatology.

[B17] Gesierich A, Herzog S, Grunewald SM, Tappe D, Brocker EB, Schon MP (2006). Eosinophilic folliculitis in a Caucasian patient: association with toxocariasis?. J Eur Acad Dermatol Venereol.

[B18] Hamidou MA, Gueglio B, Cassagneau E, Trewick D, Grolleau JY (1999). Henoch-Schonlein purpura associated with *Toxocara canis *infection. J Rheumatol.

[B19] Adame J, Cohen PR (1996). Eosinophilic panniculitis: diagnostic considerations and evaluation. J Am Acad Dermatol.

[B20] Seki M, Hiromatsu K, Kosai K, Fukuda Y, Kakugawa T, Nakamura F, Izumikawa K, Yanagihara K, Higashiyama Y, Miyazaki Y, Hirakata Y, Mukae H, Tashiro T, Kohno S (2006). [A case of toxocariasis with eosinophilic pleural effusion]. Kansenshogaku Zasshi.

